# Magnetic microparticle-polydimethylsiloxane composite for reversible microchannel bonding

**DOI:** 10.1080/14686996.2016.1140301

**Published:** 2016-02-29

**Authors:** Chia-Wen Tsao, Yueh-Pu Lee

**Affiliations:** ^a^ Department of Mechanical Engineering, National Central University, Taoyuan, ROC

**Keywords:** Microchannel, reversible bonding, magnetic microparticle, polydimethylsiloxane (PDMS)

## Abstract

In this study, an iron oxide magnetic microparticles and poly(dimethylsiloxane) (MMPs-PDMS) composite material was employed to demonstrate a simple high-strength reversible magnetic bonding method. This paper presents the casting of opaque-view (where optical inspection through the microchannels was impossible) and clear-view (where optical inspection through the microchannel was possible) MMPs-PDMS. The influence of the microchannel geometries on the casting of the opaque-view casting was limited, which is similar to standard PDMS casting. Clear-view casting performance was highly associated with the microchannel geometries. The effects of the microchannel layout and the gap between the PDMS cover layer and the micromold substrate were thoroughly investigated. Compared with the native PDMS bonding strength of 31 kPa, the MMPs-PDMS magnetic bonding experiments showed that the thin PDMS film with an MMPs-PDMS layer effectively reduced the surface roughness and enhanced MMPs-PDMS reversible magnetic bonding strength. A thin PDMS film-coated opaque-view MMPs-PDMS device exhibited the greatest bonding strength of 110 kPa, and a clear-view MMPs-PDMS device with a thin PDMS film attained a magnetic bonding strength of 81 kPa.

## Graphical abstract

**Figure:**
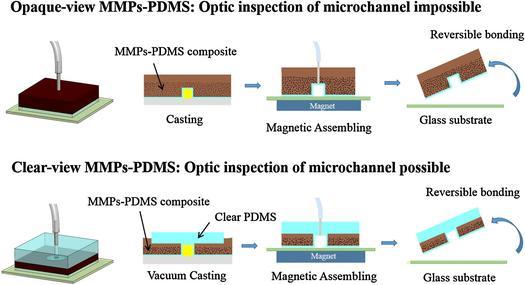

## 1. Introduction

Microfluidic, biomedical, and lab-on-a-chip devices are used for rapid, high-throughput, high-automation, and high-integration analyses, which have gained prominence in the fields of chemistry [1,2] and biology [3–5]. Various materials, such as glass [6], silicon [7], and polymers [8] are used for manufacturing microfluidic devices. Recent trends in microfluidics technology are moving toward personalized diagnosis and point-of-care-testing applications. Polymer materials are an ideal material choice for these diagnostic applications because of the low cost and disposable advantages [9]. Polymers used in microfluidics can typically be divided into two major categories: thermoplastic and thermoset polymers. Thermoplastic polymers, such as polycarbonate, poly(methyl methacrylate), and cyclic olefin copolymer (COC), are commonly used in microfluidic devices because of their superior mechanical, optical, and chemical properties. Polydimethylsiloxane (PDMS) is the most common elastomer material used in microfluidics; because of its favorable biocompatibility and gas transmissivity, PDMS has been applied extensively in cell culture applications. In addition, the success of PDMS-based microfluidic valves and pumps developed by Quake [10] has advanced PDMS microfluidics to include various applications.

Thermoplastic and PDMS polymers can be fabricated through low-cost and high-fabrication throughput polymer replication processes. Thermoplastic polymers can be reshaped while being heated above a given glass transition temperature; therefore, thermoplastic microfluidic devices are typically fabricated through hot embossing [8, 11] or inject molding [12]. PDMS is a thermoset material that is primarily used in casting for PDMS replication [13].

After polymer replication is completed, the polymer replication must be sealed using a different type of substrate layer to create an enclosed microfluidic channel. Developing an appropriate sealing method is critical for fabricating microfluidic devices. Thermoplastic polymers can be directly bonded through thermal bonding [14] by heating and pressing a pair of thermoplastic layers together. PDMS substrates can be directly attached to another PDMS substrate or glass substrate through the van der Waals force [15]. However, such microfluidic devices are prone to debonding or leaking during handling and several other types of high-pressure application. Consequently, high-strength polymer bonding methods have been developed to address these problems.

Thermoplastic bonding can be enhanced through solvent treatment [16–18] or surface modification [19,20] techniques. PDMS bonding is generally enhanced using O_2_ plasma bonding [21,22], although plasma treatment is the most common bonding technique for PDMS because of its high bond strength and simple process. Nevertheless, plasma bonding is irreversible and the surface properties are typically altered after modification. A reversible bonding method enables fabrication of dismountable and reusable microfluidic devices, which is beneficial for biomolecules analysis, cell analysis, and other related fields. Although PDMS can reversibly bond to a smooth surface through the van der Waals force, the bond strength is typically below 35 kPa [15]. Accordingly, high-strength reversible PDMS bonding methods have been developed to resolve these problems. PDMS nanocapillaries can reversibly bond to gold or silicon substrates through electrostatic forces [23]. A bioinspired method that involves using gecko-foot-mimetic nanopillar arrays coated with a mussel-adhesive-protein-mimetic polymer has also been proposed [24]. Other reversible bonding techniques involve using a direct mechanical clamping glass-PDMS-glass device [25] or vacuum aspiration [26,27], both of which achieve a high bond strength (typically above 100 kPa), but additional working space is required for the mechanical clamps or vacuum pump. Additionally, a suction microchannel network and continuous vacuuming are required during microfluidic operation when using in the vacuum aspiration method, which limits the microchannel layout and its applications.

Reversible bonding through magnetic forces is an effective alternative bonding approach. Magnetic reversible bonding is achieved by placing a microfluidic device directly on a magnet and removing it from the magnet to dismount the microchannel. Rafat et al*.* [28] assembled iron slabs around a microchannel for magnetic reversible bonding. However, because of the presence of magnetic slabs, the microchannels were restricted to simple geometries. Rasponi et al*.* [29] presented another reversible magnetic bonding method in which iron micropowders were injected or plastered into micropowder layer cavities and then align-bonded to the microchannel. This bonding method required an additional micropowder layer and precise bonding alignment or micropowder injection during the fabrication process, rendering the fabrication process complex and making it difficult to create complex parallel multiplex microchannels. In addition, iron powders usually contain large particles (maximum grain size: 212 μm), which restricted the minimum microchannel width of the micropowder cavity layer. In their research demonstration, the minimum microchannel width in cavity layer is 800 μm. Thus, both the iron slab and micropowder approaches can provide magnetic sealing only near the major fluidic pathway instead of fully sealing each branch-microchannel boundary for complete sealing.

In this study, we first demonstrate the use of synthesized magnetic iron oxide (Fe_3_O_4_) microparticles and an MMPs-PDMS composite for reversible magnetic bonding applications. This paper presents the procedure for casting opaque-view (where the optical inspection of the microchannel was impossible) and clear-view (where the optical inspection of the microchannel was possible) MMP-PDMSs. The grain size of the synthesized MMPs is <27 μm, and the MMPs-PDMS composite directly fills the micromold cavity during the casting procedure, completely sealing each microchannel without requiring precision alignments and assembly during the casting procedure. Additionally, MMPs-PDMS casting and its effects on reversible magnetic bonding strength are evaluated in this paper in detail.

## 2. Experiment

### 2.1. Materials

P-type (100) silicon wafer with diameter of 10 cm was purchased from Summit-Tech Resource Corp. (Hsinchu, Taiwan). SU-8 3050 and SU-8 developer were purchased from MicroChem Corp. (Newton, MA, USA), and PDMS (Sylgard 184 silicone elastomer kit) was purchased from Dow Corning Corp. (Midland, MI, USA). Acetone and isopropanol were purchased from J.T Baker (Phillipsburg, NJ, USA). Surgical needles (SC20/15, LS20) were purchased from Instech Laboratories, Inc. (Plymouth Meeting, PA, USA). Medical grade tubing (Tygon S-50-HL) was purchased from Saint Gobain Performance Plastics Corp. (Aurora, OH, USA). Ammonia was purchased from Merck Co. (Darmstadt, Germany). Hydrochloric acid (HCl), iron (II) chloride tetrahydrate (FeCl_2_·4H_2_O), and iron (III) chloride hexahydrate (FeCl_3_·6H_2_O) were purchased from Sigma-Aldrich (St. Louis, MO, USA).

2.2. 

Iron oxide magnetic microparticle synthesis

Fe_3_O_4_ MMPs were fabricated using a magnetic nanoparticle synthesis procedure [30]; 2.0 g of FeCl_2_·4H_2_O and 5.4 g of FeCl_3_·6H_2_O were mixed with 25 ml of a 2 M HCl solution and degassed using ultrasonication. Subsequently, 25 ml of 25% (v/v) ammonia was added and stirred for 30 min at room temperature. A magnet was placed under the beaker to concentrate the Fe_3_O_4_ particles and completely rinsed with deionized water. The Fe_3_O_4_ was baked at 150°C for 4 h on a hotplate to remove the water from the MMPs in order to prevent bubbles forming during vacuum casting because of the MMPs, which would create voids after the MMPs-PDMS layer was cured. Finally, the dry clodded MMPs were finely ground using a mesh sieve to create MMPs 4–27 µm in size.

### 2.3. SU-8 micromold fabrication

**Figure 1.**
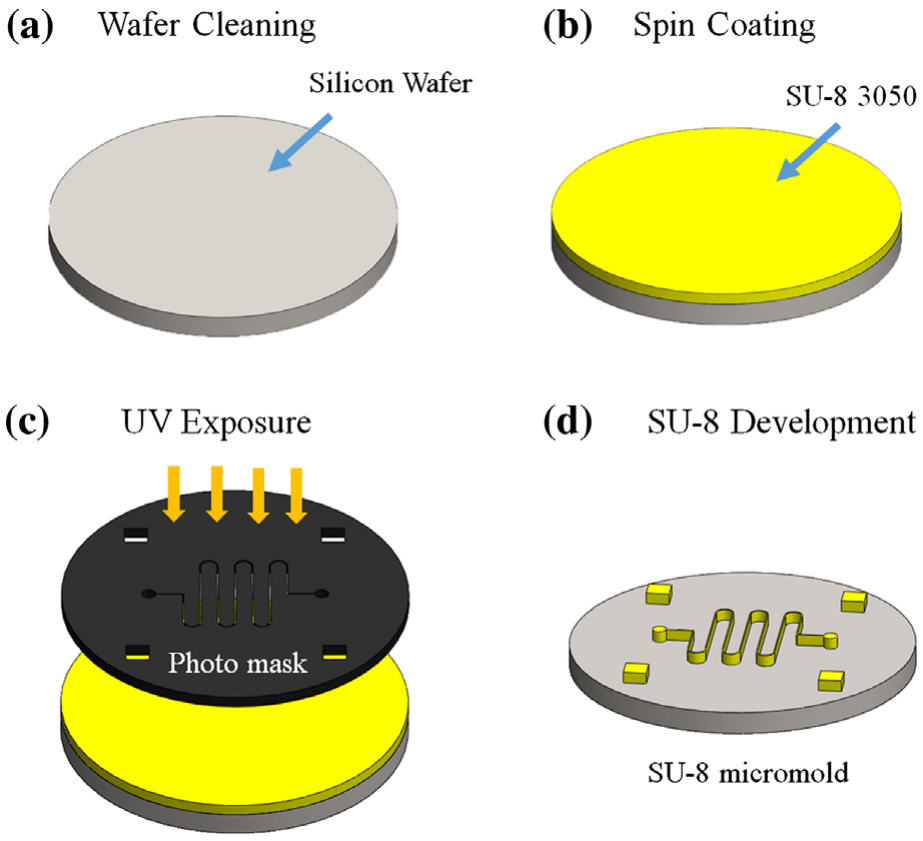
Schematic illustration of SU-8 micromold fabrication process: (a) wafer cleaning; (b) spin coat SU-8 photoresist; (c) UV exposure; and (d) development of SU-8 to create a micromold.

The micromold was fabricated using SU-8 lithography process (Figure 1). First, a silicon wafer was cleaned with acetone, isopropanol, and deionized water in a sonicator, followed by drying with a N_2_ gun and hot-baking at 130C for 15 min (Super-Nuova, Thermo Scientific, Inc., Waltham, MA, USA) to remove any surface moisture. Second, a two-step spinning process was performed at 500 rpm for 30 s and at 1000 rpm for 40 s (SPC-703, Yi Yang Co., Taoyuan, Taiwan) to coat a SU-3050 photoresist layer onto the silicon wafer, which was then soft-baked at 110C for 20 min. The photomask was aligned and the SU-8-coated wafer was exposed to UV radiation (AGL100 UV Light source, M&R Nano Technology Co., Taoyuan, Taiwan) for 90 s. Subsequently, the wafer was soft-baked at 95C for 5 min and then rinsed with the SU-8 developer to create a 100-μm-thick SU-8 micromold. The SU-8 coating steps (Figure 1(b)) were repeated to create a 200-μm-thick SU-8 micromold.

### 2.4. Magnetic microparticles-poly(dimethylsiloxane) composite preparation

The PDMS mixture was prepared by first mixing the PDMS base and a curing agent at a 10:1 weight ratio, and then fully mixing it with the MMPs powder at a weight ratio of 1:10, 1:5, and 2:5 to obtain the MMPs-PDMS composites. The MMPs-PDMS composite was degassed in a vacuum desiccator at a gauge pressure of <700 mm Hg (PC-250, Yeong-shin, Hsinchu, Taiwan) to remove the air bubbles from the composite.

### 2.5. Cover-PDMS layer preparation

The cover-PDMS layer was fabricated using a standard PDMS lithography process [31]. The vacuum-degassed 1:10 PDMS mixture was poured onto the silicon wafer by using a 3-mm-thick spacer and any excessive PDMS mixture was removed. Subsequently, the mixture was baked at 60°C for 3 h and cut-removed, yielding an optically clear PDMS as a cover layer for use in the clear-view MMPs-PDMS casting procedure.

## 3. Results and discussion

**Figure 2.**
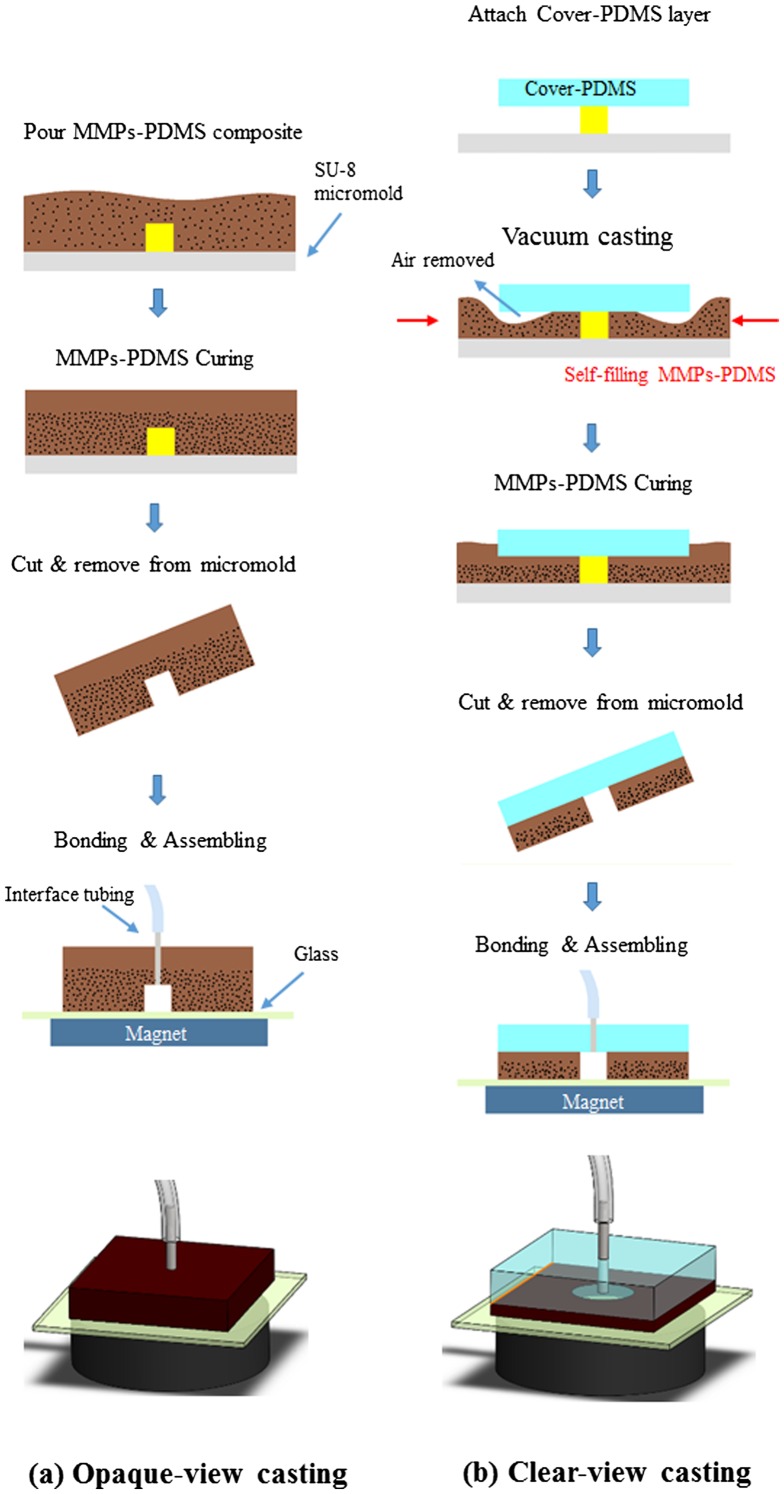
Schematic illustration of the (a) opaque-view and (b) clear-view MMPs-PDMS casting procedures.

Figure 2 illustrates the opaque- and clear-view MMPs-PDMS layer casting procedures. The opaque-view MMPs-PDMS layer casting (Figure 2(a)) was fabricated using a standard PDMS process. In this process, the MMPs-PDMS composite was poured into the SU-8 micromold and cured on a hot plate at 60°C for 4 h to create the 3-mm-thick MMPs-PDMS layer. This process was simple and straightforward for creating an MMPs-embedded PDMS layer. However, the opaque-view MMPs-PDMS layer was completely covered with dark MMPs, which blocked the microchannel, rendering optical inspection impossible. This could introduce problems in some optical-based microfluidic applications.

Creating a clear top-view PDMS microchannel surrounded by the MMPs for reversible magnetic bonding purposes is a complex procedure. Multistep casting and bonding approaches are impractical because they require precise alignment and assembling. Cast-scraping, which involves removing the MMPs-PDMS mixture followed by casting another optically clear PDMS layer above the MMPs-PDMS layer, at first seems to be a feasible clear-view MMPs-PDMS fabrication method. However, using a thin MMPs-PDMS layer above the SU-8 micromold after the scraping step partially blocks the optical view in the microchannel after the PDMS has been cured. This is because of the behavior of the highly viscous MMPs-PDMS composite. Several adhesive microfluidic bonding techniques involve utilizing this viscous property to create a thin adhesive layer for adhesive bonding [32].

In this study, we propose a simple clear-view MMPs-PDMS casting procedure (Figure 2(b)) for creating an optically inspectable microchannel. First, the 3-mm-thick PDMS cover layer was attached to the SU-8 micromold, and the MMPs-PDMS composite was applied around the cover-PDMS layer. The micromold was placed in a vacuum desiccator with gauge pressure of <700 mm Hg and baked at 60C for 4 h. The MMPs-PDMS composite was adequately integrated with the PDMS cover layer, and no breakage was observed at the interface after the MMPs-PDMS casting. The MMPs-PDMS layers were pierced with 0.75-mm-diameter holes and surgical needles were inserted to form microchannel inlets and outlets. The MMPs-PDMS microchannel layer was attached to a glass substrate and assembled with a 5450 gauss magnet (diameter: 4 cm; thickness: 2 cm) as a reversible-bond microfluidic device.

**Figure 3.**
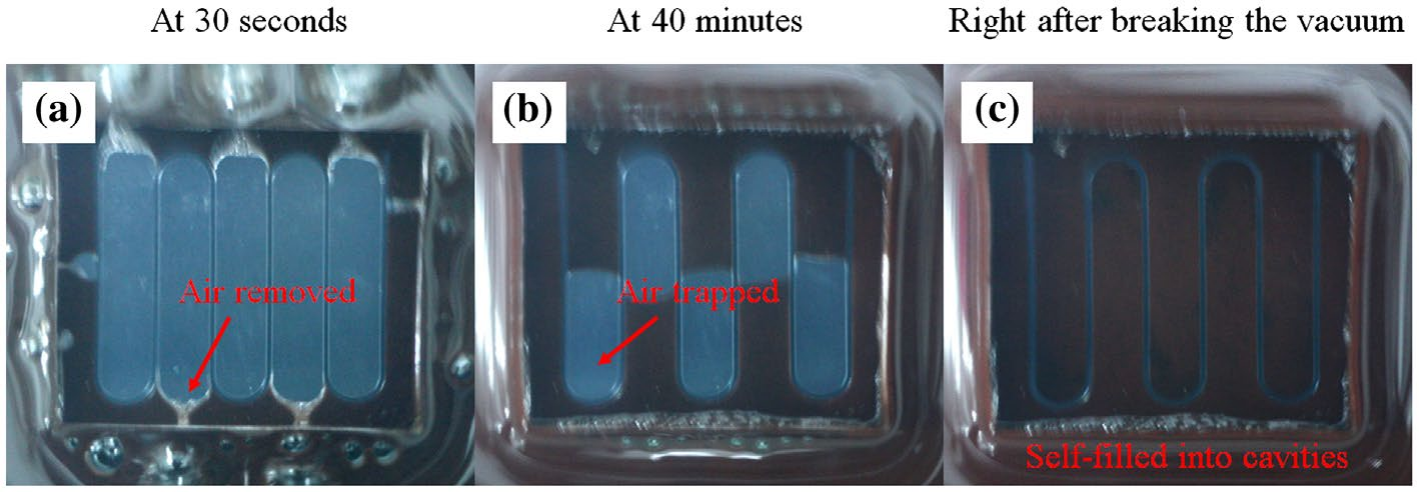
Vacuum casting steps in the serpentine clear-view casting process (a) at 30 s; (b) at 40 min; and (c) after breaking the vacuum.

Figure 3 shows consequent images of the opaque-view MMPs-PDMS vacuum-casting procedure, which involved removing air trapped from the intermediate layer and allowing the MMPs-PDMS composite to flow into the intermediate layer cavities to form the clear-view MMPs-embedded PDMS microchannel layer. As shown in Figure 3(a), when the vacuum was turned on, the air in the gap between the PDMS cover layer and the micromold was immediately removed. For simple open-cell geometries (e.g. straight microchannels or circular microfluidic chambers), the air can easily be removed and the gap in the MMPs-PDMS composite can be filled without trapping air. However, for several types of complex closed-cell geometries, such as the U-shaped serpentine microchannels, air becomes trapped in the U-turn sections of the microchannel (Figure 3(b)). However, because PDMS is a gas permeable material, the trapped air can gradually be removed through the PDMS membrane. When the vacuum was broken (Figure 3(c)), the MMPs-PDMS composite had completely self-filled the micromold cavities.

### 3.1. Microchannel geometry effects on MMPs-PDMS casting

In both the opaque- and clear-view MMPs-PDMS casting, the amount of MMPs embedded in the PDMS layer affected the reversible magnetic bonding strength, which depends on the concentration of the MMPs-PDMS composite. In addition, the layout of the microchannel may also affect the distribution of the MMPs embedded in the PDMS layer, as well as its reversible magnetic bonding performance. Therefore, this study investigated the MMPs-PDMS composite concentration and microchannel geometry effects to opaque- and clear-view MMPs-PDMS microchannel casting. Three MMPs-PDMS composite concentrations of 1:10, 1:5, and 2:5 (MMPs:PDMS weight ratio) and three general microchannel geometries (i.e. straight, serpentine, and zigzag) were evaluated. The zag microchannel geometries were 100 μm wide and 10 mm long, with four length-to-space aspect ratios of 5 (space: 2 mm), 10 (space: 1 mm), 25 (space: 0.4 mm), and 50 (space: 0.2 mm).

#### 3.1.1. Opaque-view microchannel casting

**Figure 4.**
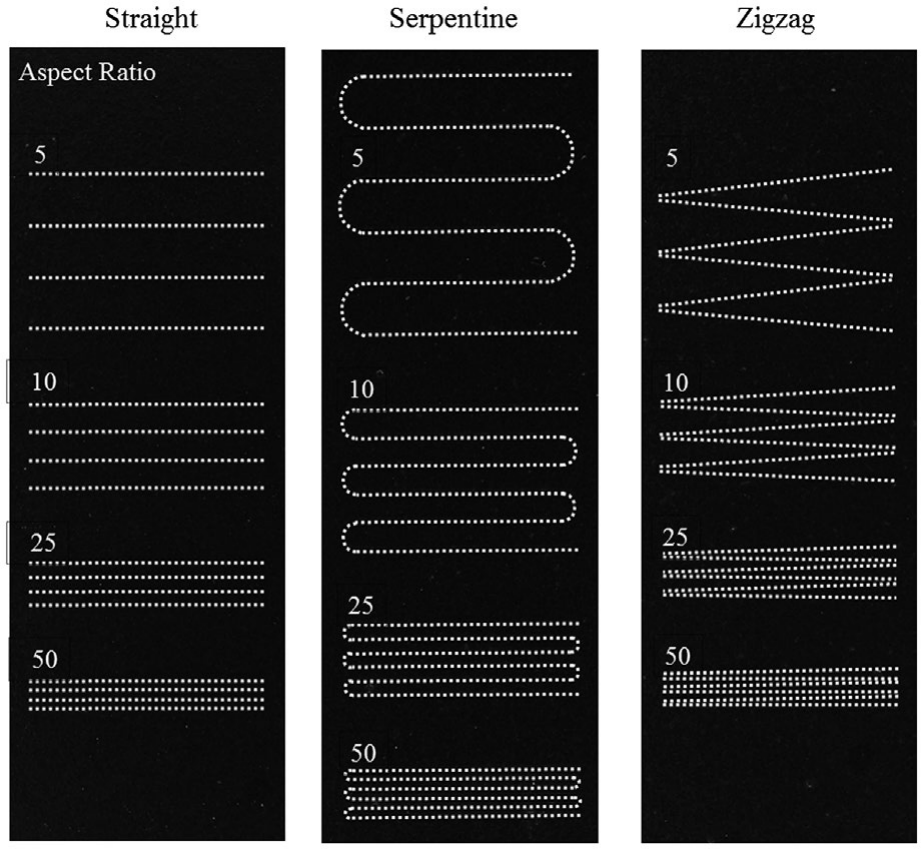
Opaque MMPs-PDMS casting of (a) straight; (b) serpentine; and (c) zigzag microchannels with aspect ratios of 5, 10, 25, and 50. White dot lines are used to indicate the microchannels because they cannot be clearly seen in the opaque-view MMPs-PDMS layer.

Opaque-view MMPs-PDMS casting is a simple procedure that is similar to standard PDMS casting. The MMPs-PDMS composite was successfully cast on a 100-μm-thick microchannel mold for all test geometry and concentration conditions. Figure 4 shows the opaque-view MMPs-PDMS straight, serpentine, and zigzag microchannel casting results with a 2:5 MMPs-PDMS concentration. Uniform MMPs were embedded in the PDMS layer for all geometry conditions.

#### 3.1.2. Clear-view straight microchannel casting

**Figure 5.**
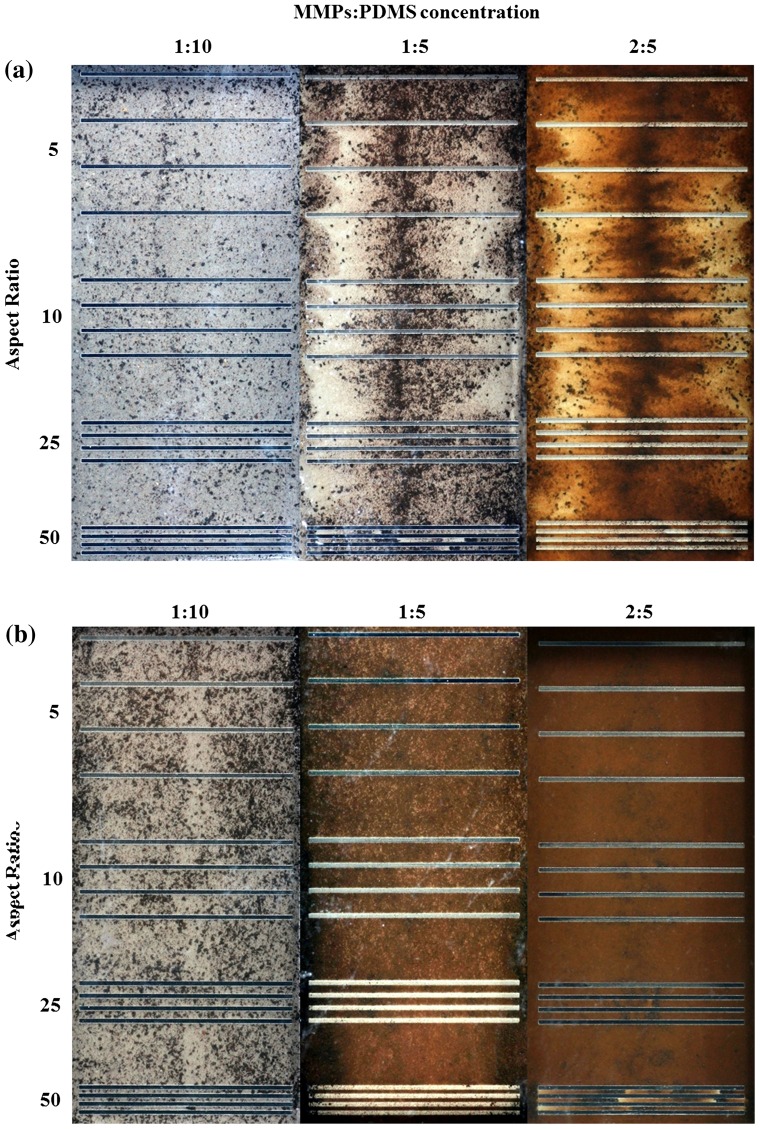
Clear-view MMPs-PDMS casting of (a) 100-μm-thick and (b) 200-μm-thick straight microchannels with composite concentrations of 1:10, 1:5, and 2:5 at aspect ratios of 5, 10, 25, and 50.

The microchannel geometries were independent in the opaque-view casting because the MMPs-PDMS composite could move freely into the micromold cavities and remove air bubbles, such as in standard PDMS casting. However, the clear-view casting performance was closely related to the microchannel geometries. The geometrical constraints of the microchannel layout and gap between the PDMS cover layer and micromold substrate could result in trapped air bubbles or an irregular distribution of MMPs after MMPs-PDMS curing. Therefore, the clear-view straight microchannels were evaluated first; Figure 5 shows the casting results obtained from the various MMPs-PDMS composite concentrations.

The MMPs-PDMS composite successfully filled the straight microchannel for the designs with a height of 100 μm (Figure 5(a)) and 200 μm (Figure 5(b)). The MMPs-PDMS layer became darker as the MMPs-PDMS composite became more concentrated, indicating that more MMPs were embedded in the PDMS layer at higher concentrations. No trapped air bubbles were observed in any condition in the straight microchannels; however, an irregular MMPs distribution was observed in the 100-μm-thick microchannel case (Figure 5(a)), where MMPs were more concentrated at the center of the microchannel. This occurred because the mixture was injected through the openings at both ends of the microchannel, merging in the center. When the gap between the PDMS cover layer and the micromold substrate was limited, MMPs accumulated and concentrated at the center. In the 200-μm-thick microchannel condition (Figure 5(b)), a regular distribution of MMPs was observed because the wider gap between the PDMS cover layer and SU-8 substrate allowed the MMPs to move more freely during the casting process. An irregular MMPs distribution was occasionally observed under the high aspect ratio conditions (i.e. 50). This irregular distribution can result in an uneven distribution of magnetic bonding strength, causing local weak bonding, which can create local leakage during microfluidic operation.

#### 3.1.3. Clear-view serpentine microchannel casting

**Figure 6.**
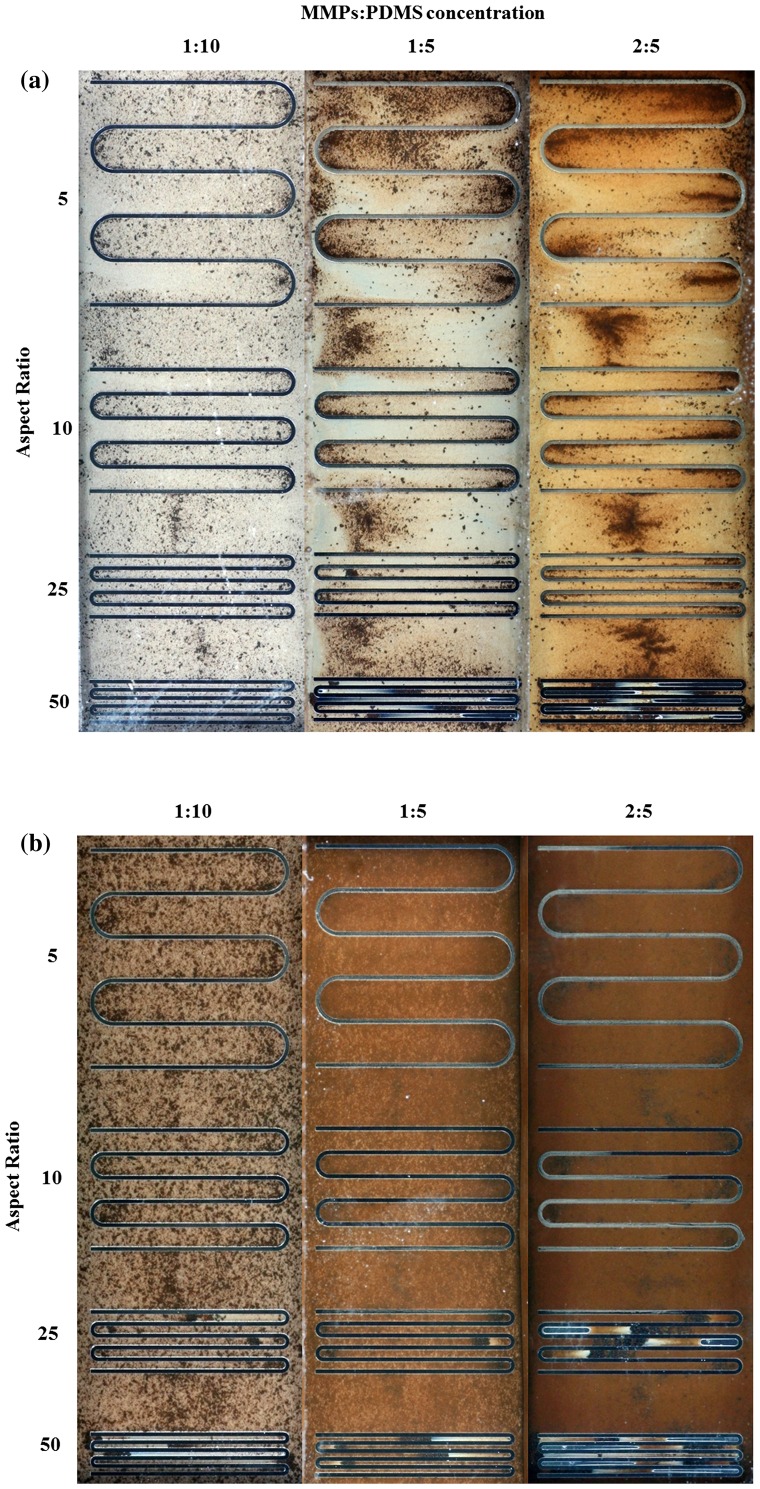
Clear-view MMPs-PDMS casting of (a) 100-μm-thick and (b) 200-μm-thick serpentine microchannels with composite concentrations of 1:10, 1:5, and 2:5 at aspect ratios of 5, 10, 25, and 50.

In microfluidic applications, serpentine or U-shaped microchannels are a commonly used to minimize a device’s footprint. Figure 6(a) shows the 100-μm-thick serpentine microchannel MMPs-PDMS casting results from this study. An irregular MMPs distribution was observed at all 1:10, 1:5, and 2:5 concentrations. During the vacuum casting procedure, the MMPs-PDMS composite filled the opening of the U-shaped cavities, causing the MMPs to accumulate at the U-shaped ends, filling the entire height of the 100-μm-thick microchannel. Compared with the 200-μm-thick microchannel condition (Figure 6(b)), the MMPs were more evenly distributed, indicating that the MMPs were less geometrically constrained during casting when taller microchannels were used.

Trapped air was observed in the serpentine microchannel layout. During the casting process, air in the enclosed U-shaped pocket could not be expelled effectively, resulting in air bubbles forming in the high-aspect-ratio MMPs-PDMS layers after curing. As shown in Figure 6, trapped air was observed in the U-shaped section of the layers with aspect ratios of 25 and 50.

#### 3.1.4. Clear-view zigzag microchannel casting

**Figure 7.**
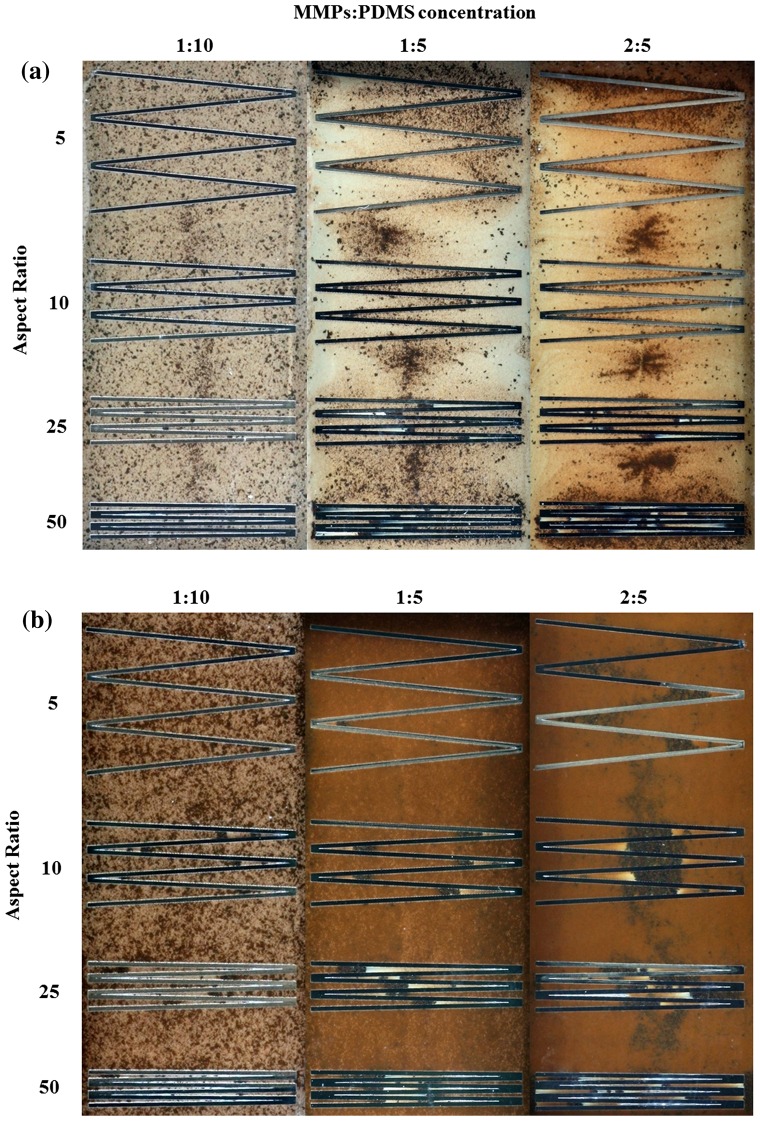
Clear-view MMPs-PDMS casting of (a) 100-μm-thick and (b) 200-μm-thick zigzag microchannels with composite concentrations of 1:10, 1:5, and 2:5 at aspect ratios of 5, 10, 25, and 50.

Microfluidic devices with zigzag microchannels feature sharp edges or turns in the microchannel design. The zigzag microchannel casting results (Figure 7) are similar to those for the serpentine microchannel design; the MMPs tended to accumulate in the zigzag corners in the 100-μm-thick microchannel (Figure 7(a)), whereas the distribution was more even in the 200-μm-thick microchannel (Figure 7(b)). During the casting process, the sharp zigzag edges were more difficult to expel air from compared with the straight and U-shaped serpentine microchannels. Trapped air bubbles were more frequently observed in the high-aspect ratios of 25 and 50.

### 3.2. MMPs-PDMS reversible bonding

**Figure 8.**
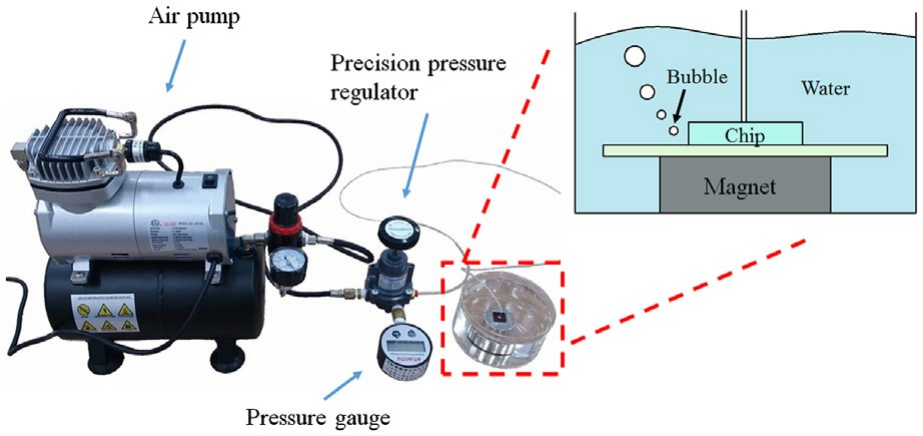
Schematic illustration of bonding experiment setup.

Figure 8 shows the reversible bonding test experiment setup. The magnetically assembled MMPs-PDMS microfluidic device was placed in a beaker filled with water. An air pump with a precision pressure regulator (Type 400, Control Air, Inc., Amherst, NH, USA) and pressure gauge (DPG-M2.5, Atlantis Ltd, Taipei, Taiwan) was connected to the MMPs-PDMS microfluidic device through a 5-mm-diameter enclosed microfluidic chamber designed for the bonding test. The air pump pressure was initially set at 0 kPa and increased by 2–3 kPa every 5 s. The values on the pressure gauge were recorded as the maximal bonding strength while air bubbles were leaking out of the microchannel. The MMPs-PDMS and glass surfaces were carefully cleaned and dried to ensure that no liquid film or surface contaminated the interface before each reversible bonding test.

**Figure 9.**
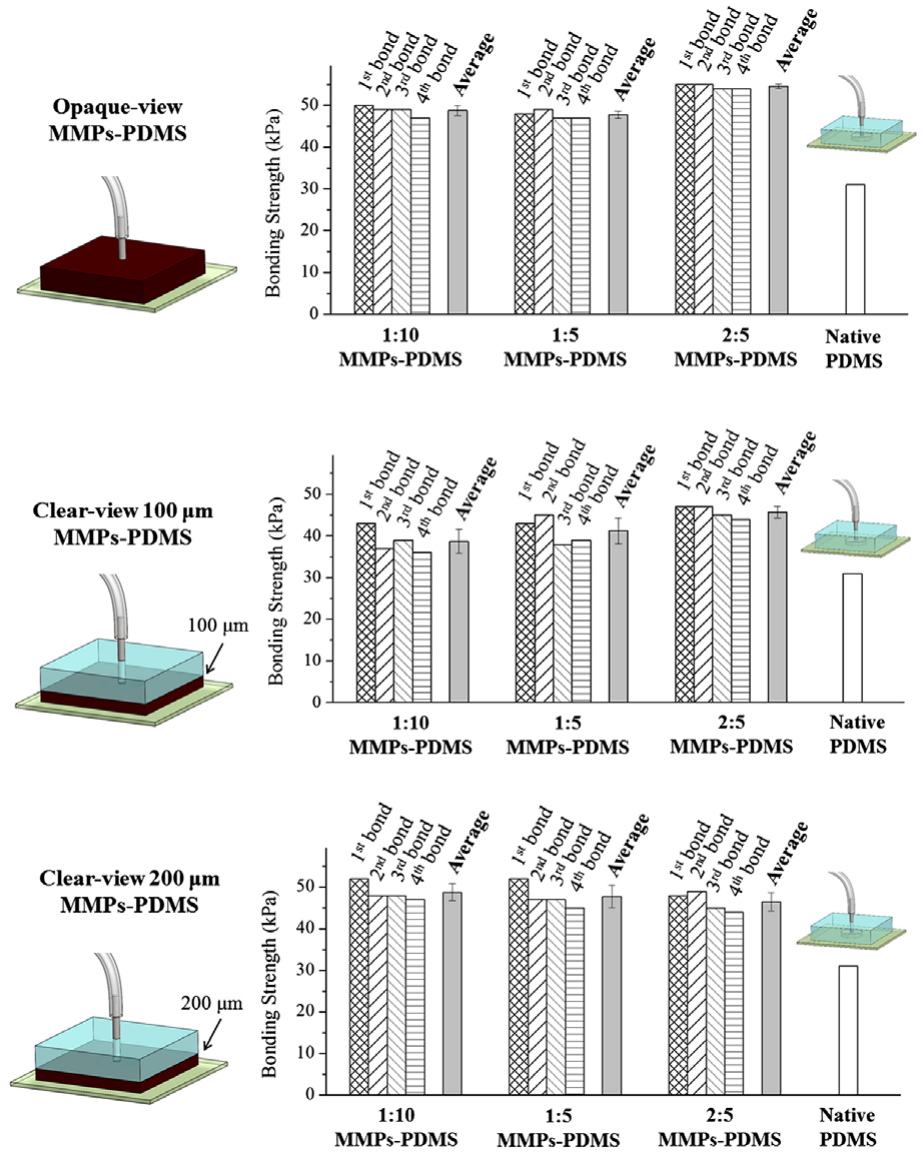
Bonding strength of the MMPs-PDMS microchannel.

Figure 9 shows the average and reversible bonding strength of the opaque- and clear-view MMPs-PDMS composites with concentrations of 1:10, 1:5, and 2:5. The maximum MMPs-PDMS concentration of 2:5 was selected because the MMPs-PDMS layer cracked at higher concentrations. The native PDMS-glass bonding strength was 31 kPa, which is comparable with another study (35 kPa) [15]. All bonding strength measurements of the opaque-view MMPs-PDMS were between 47 and 55 kPa. In the clear-view reversible bonding, the bonding strength was measured between 38 and 46 kPa for the 100-μm-thick microchannel and between 48 and 55 kPa for the 200-μm-thick microchannel. From the reversible bonding results (Bonds 1 to 4), as shown in Figure 9, the magnetic reversible bonding process was highly reversible because the MMPs-PDMS bonding strength was primarily created by the van der Waals adhesion from the MMPs-PDMS/glass interface and the magnetic force from the MMPs embedded in the MMPs-PDMS layer. However, we still observed an average 2.7% (maximum 13.9%) reduction trend, which might have been caused by surface damage or contamination at the bonding interface after each reversible bond, despite the surface cleaning and drying between each test.

According to the MMPs-PDMS bonding results (Figure 9), the bonding strength was enhanced only approximately 0.5-fold, and few correlations were observed between the bonding strength and MMPs concentrations. For example, for the 200-μm-thick clear-view reversible bonding (Figure 9), we observed a decrease in bonding strength when the MMPs concentration was increased. We attributed the weak correlations between the bonding strength and process parameters and the weak bonding strength enhancement to the rough surface at the MMPs-PDMS/glass substrate interface. Our MMPs-PDMS casting experiments revealed that the MMPs precipitated at the bottom layer during the vacuum casting procedure, and the MMPs accumulating in the bottom layer affected the flatness of the MMPs-PDMS surface. During the reversible MMPs-PDMS magnetic bonding process, the MMPs-PDMS layer was elastically deformed by magnetic forces and became attached to the glass substrate, achieving conformity at the bonding interface. When the MMPs-PDMS surface was not adequately flat, the bonding strength from the magnetic force was reduced, resulting in an overall reduction in bonding strength.

**Figure 10.**
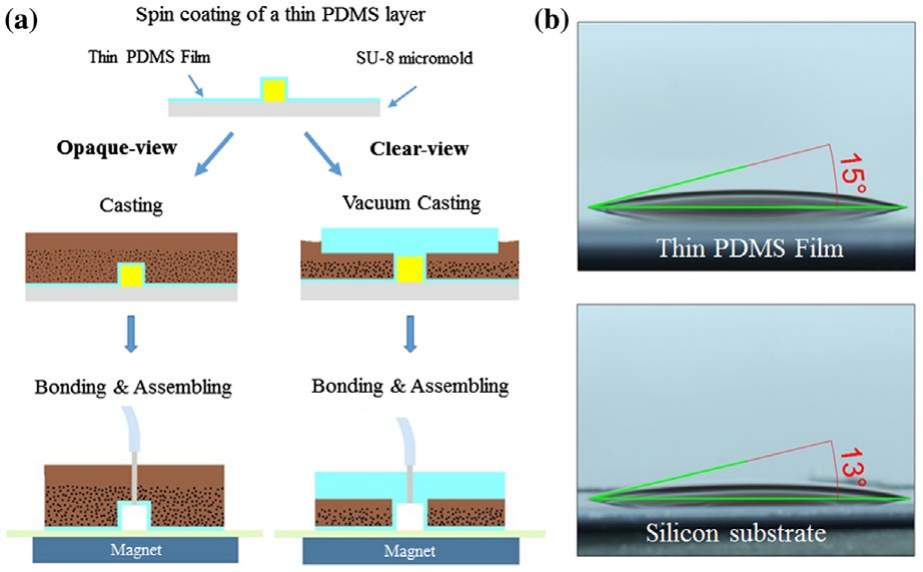
(a) MMPs-PDMS casting procedure with thin PDMS film. (b) Surface wettability of a 2:5 concentration MMPs-PDMS composite droplet on a thin PDMS film and on a silicon substrate.

To solve this problem, we spin-coated (at 1800 rpm for 40 s) a 50-μm-thick PDMS layer onto the SU-8 micromold substrate prior to MMPs-PDMS casting, thereby minimizing the roughness of the MMPs-PDMS bottom surface. Figure 10(a) shows the opaque- and clear-view MMPs-PDMS casting procedures with the thin PDMS films. The thin PDMS film-coated MMPs-PDMS casting, bonding, and assembling procedures are analogous to the previously described MMPs-PDMS casting procedure. The only difference is that a thin PDMS film was present at the bottom of the micromold during the casting procedure. Figure 10(b) shows the surface wettability of the 2:5 MMPs-PDMS composite droplet on a thin PDMS film and on a silicon substrate. The MMPs-PDMS composite contact angles were 15° on the PDMS thin film and 13° on the silicon substrate. These results show that the surface wettability of the silicon or PDMS film-coated silicon substrates had a limited effect during the casting procedure.

**Figure 11.**
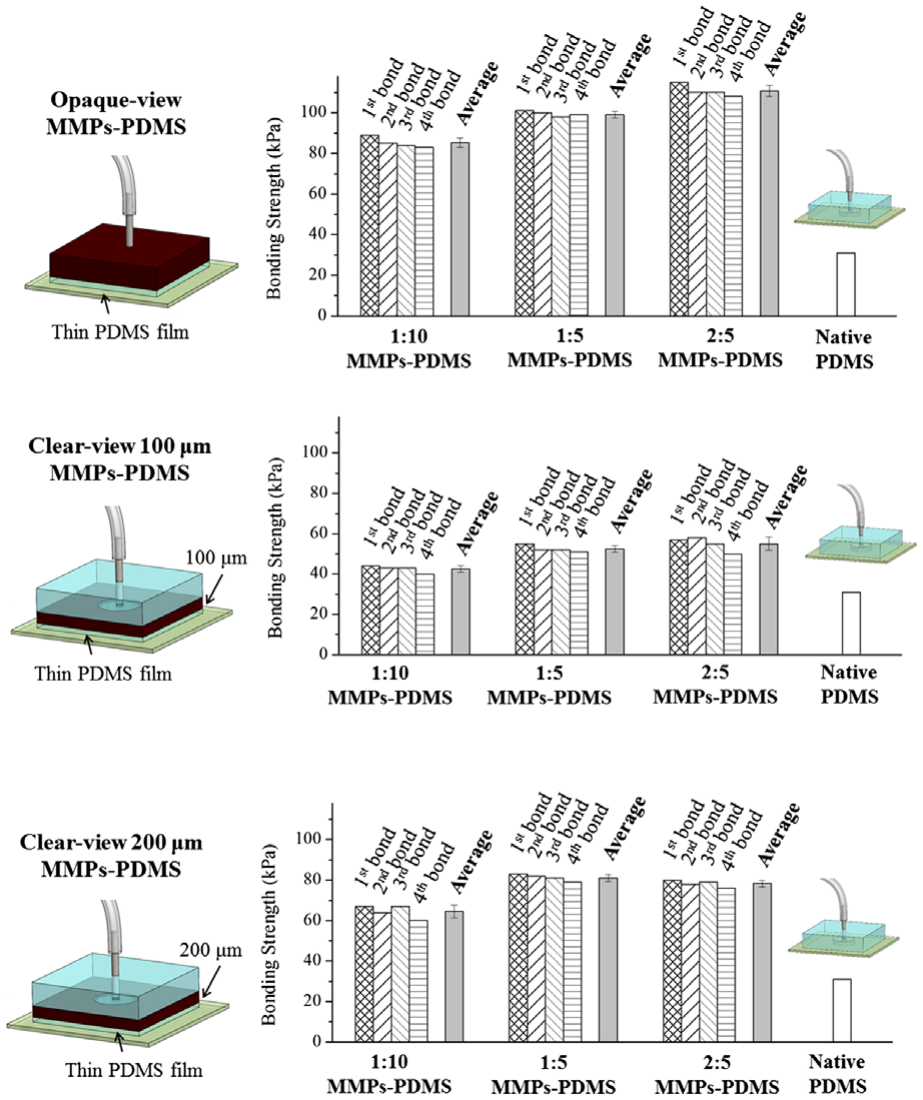
Bonding strength of MMPs-PDMS microchannel with thin PDMS film coating.

Figure 11 shows the average and reversible bonding strength of the MMPs-PDMS layers with the thin PDMS films. The opaque-view MMPs-PDMS bonding strength was measured at 85.3 ± 2.4 kPa (1:10), 99.0 ± 1.7 kPa (1:5), and 110.8 ± 2.8 kPa (2:5). The bond strength increased with the MMPs-PDMS composite concentration, and the maximum bonding strength enhancement was approximately 3.5-fold that of the bonding of native PDMS. For the clear-view bonding experiments, the bonding strength was 42.5 ± 1.6 kPa (1:10), 52.5 ± 1.6 kPa (1:5), and 55.0 ± 3.3 kPa (2:5) for the 100-μm-thick clear-view MMPs-PDMS layer and 64.5 ± 3.1 kPa (1:10), 81.0 ± 1.7 kPa (1:5), and 78.3 ± 1.6 kPa (2:5) for the 200-μm-thick clear-view MMPs-PDMS layer. The 200-μm-thick microchannels exhibited a greater enhancement in bonding strength compared with the 100-μm-thick microchannels because the active magnetic MMPs-PDMS layer was thicker and more uniformly distributed in the PDMS layer. The maximum bonding strength enhancement of the clear-view bonding process was approximately 2.8-fold that of the native PDMS bonding. Good bonding reversibility was also observed in the thin PDMS-coated MMPs-PDMS device. On average, the bonding strength exhibited a 2.4% reduction trend (maximum 10.4%) with each reversible bonding test.

**Figure 12.**
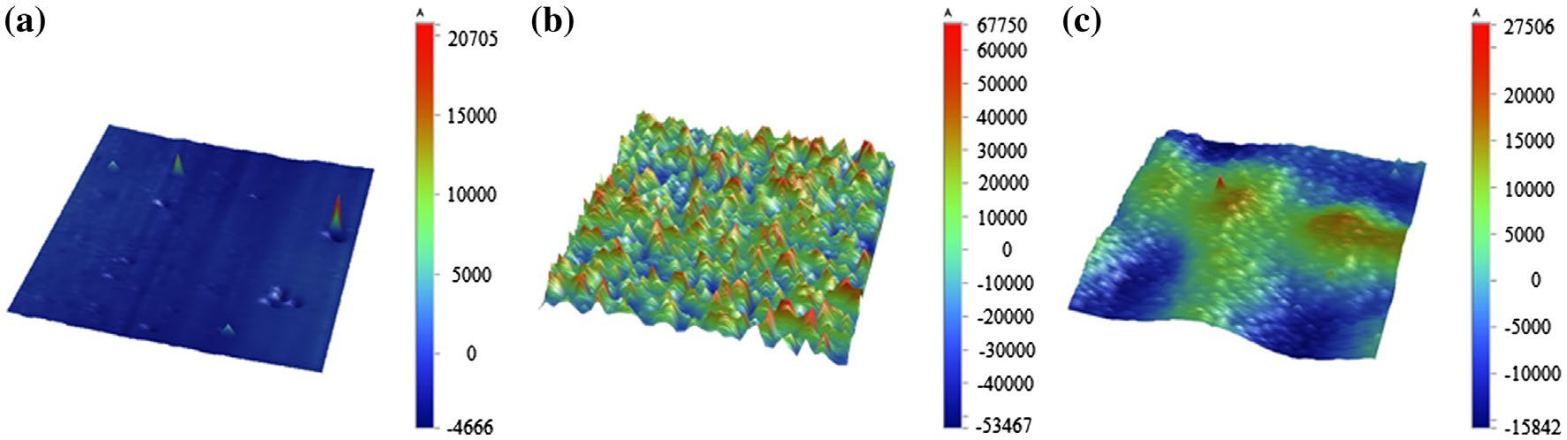
Surface roughness measurement of 1 × 1 mm (a) native PDMS, (b) 2:5 concentration MMPs-PDMS layer, and (c) 2:5 concentration MMPs-PDMS layer with thin PDMS film on the bottom.

The bonding strength of MMPs-PDMS can effectively be improved by using thin PDMS films. Figure 12 shows measurements taken with a surface profilometer (Dektak XT, Bruker, Billerica, MA, USA) of native PDMS (Figure 12(a)), a 2:5 MMPs-PDMS layer without the thin PDMS film (Figure 12(b)) and with the thin PDMS film (Figure 12(c)). The native PDMS surface was the smoothest surface (average roughness: 308 Å), and the 2:5 MMPs-PDMS layer exhibited a considerably rougher surface (average roughness: 16456 Å). With a thin PDMS film covering the 2:5 MMPs-PDMS layer, the surface became flatter (average roughness: 1397 Å). These results show that the surface roughness at the MMPs-PDMS/glass bonding interface was critical to the overall bonding strength. The MMPs-PDMS layer without the PDMS thin film (Figure 9) exhibited high surface roughness, which explains why the MMPs-PDMS layer without the thin PDMS film attained a bonding strength enhancement of only 50% compared with the layer with the thin film (in presence of magnetic forces). Although an additional thin PDMS film on the MMPs-PDMS surface can minimize the roughness effect and enhance the reversible MMPs-PDMS bonding strength, the effects from variation in the surface roughness persisted. The surface of the thin PDMS-coated MMPs-PDMS layer remained rougher than the native PDMS. Therefore, there is an optimal bonding strength for the 200-μm-thick 1:5 concentration clear-view MMPs-PDMS layer (81.0 kPa), and for the 2:5 concentration opaque-view MMPs-PDMS layer (110.8 kPa).

## 4. Conclusions

An Fe_3_O_4_ magnetic MMPs-PDMS composite was successfully used to demonstrate the effectiveness of a simple high-strength reversible magnetic bonding method. In this study, the proposed method was used to cast opaque-view (where an optical inspection is impossible) and clear-view (where an optical inspection is possible) MMPs-PDMS structures. The results show that microchannel geometries have limited influence on opaque-view casting because the MMPs-PDMS composite can freely fill the micromold cavities to remove air bubbles, which is similar to standard PDMS casting. On the other hand, clear-view casting performance was closely related to the microchannel geometries. The geometrical constraints of the microchannel layout as well as the gap between the PDMS cover layer and the micromold substrate caused air bubbles to become trapped or the MMPs distribution to become irregular at high-aspect ratios.

MMPs-PDMS magnetic bonding experiments showed that MMPs-PDMS composites facilitated a highly reversible bonding process because the MMPs-PDMS bonding strength was achieved through van der Waals adhesion and magnetic forces. A thin PDMS film-coated MMPs-PDMS layer can effectively reduce the surface roughness and enhance MMPs-PDMS magnetic bonding strength. The thin PDMS film-coated opaque-view MMPs-PDMS device attained the maximum bonding strength of 110 kPa in an MMPs-PDMS composite with a 2:5 concentration. For the clear-view MMPs-PDMS devices with a thin PDMS film, a reversible magnetic bonding strength as high as 80 kPa was achieved in the 1:5 and 2:5 MMPs-PDMS composites while providing an optically inspectable microchannel. We have successfully tested the MMPs-PDMS device in microfluidic cell culture application and the MMPs-PDMS layer may also be used as magnetic-based actuator as future work.

## Notes on Contributors

Chia-Wen Tsao is Associate Professor in Department of Mechanical Engineering, in National Central University, Taoyuan, Taiwan. He obtained his Ph.D. degree in Department of Mechanical Engineering, University of Maryland at College Park, USA in 2008. After graduation, he join Department of Mechanical Engineering, National Central University, Taiwan as Assistant Professor in 2008. His research interest includes polymer and silicon micro/nano-fabrication, micro/nano-fluidics, mass spectrometry analysis and interfacing, and lab-on-chips devices.

Yueh-Pu Lee is master degree graduate school student in Department of Mechanical Engineering, in National Central University, Taoyuan, Taiwan. He received his master degree for National Central University in 2015. His research topic is polymer microfabrication process.

## Disclosure statement

No potential conflict of interest was reported by the authors.
